# Human Skin Microbiome: Impact of Intrinsic and Extrinsic Factors on Skin Microbiota

**DOI:** 10.3390/microorganisms9030543

**Published:** 2021-03-05

**Authors:** Krzysztof Skowron, Justyna Bauza-Kaszewska, Zuzanna Kraszewska, Natalia Wiktorczyk-Kapischke, Katarzyna Grudlewska-Buda, Joanna Kwiecińska-Piróg, Ewa Wałecka-Zacharska, Laura Radtke, Eugenia Gospodarek-Komkowska

**Affiliations:** 1 Department of Microbiology, Nicolaus Copernicus University in Toruń, L. Rydygier Collegium Medicum in Bydgoszcz, 85-094 Bydgoszcz, Poland; z.kraszewska@cm.umk.pl (Z.K.); natalia12127@gmail.com (N.W.-K.); katinkag@gazeta.pl (K.G.-B.); j.kwiecinska@cm.umk.pl (J.K.-P.); gospodareke@cm.umk.pl (E.G.-K.); 2 Department of Microbiology and Food Technology, UTP University of Science and Technology, 85-029 Bydgoszcz, Poland; justynabauza@gazeta.pl; 3 Department of Food Hygiene and Consumer Health, Wrocław University of Environmental and Life Sciences, 31 C.K. Norwida St., 50-375 Wrocław, Poland; ewa.walecka@upwr.edu.pl; 4 Faculty of Civil and Environmental Engineering and Architecture, UTP University of Science and Technology in Bydgoszcz, Al. prof. S. Kaliskiego 7, 85-796 Bydgoszcz, Poland; laura021@interia.pl

**Keywords:** skin, microbiome, internal factors, external factors, disinfection

## Abstract

The skin is the largest organ of the human body and it protects the body from the external environment. It has become the topic of interest of researchers from various scientific fields. Microorganisms ensure the proper functioning of the skin. Of great importance, are the mutual relations between such microorganisms and their responses to environmental impacts, as dysbiosis may contribute to serious skin diseases. Molecular methods, used for microorganism identification, allow us to gain a better understanding of the skin microbiome. The presented article contains the latest reports on the skin microbiota in health and disease. The review discusses the relationship between a properly functioning microbiome and the body’s immune system, as well as the impact of internal and external factors on the human skin microbiome.

## 1. Introduction

The skin is the largest organ of the human body, with an average surface area of 30 m^2^ in adults [1]. As the outer layer of the body—with a thickness of 2–3 mm—it functions as both a physical barrier, protecting its interior against the negative influence of various environmental factors, and an immunological barrier, reducing the effects of injuries and infections. In addition to its protective role, the skin is also responsible for thermoregulation processes, preventing water loss from the body, enabling temperature sensations, and supporting vitamin D synthesis [2,3,4,5,6].

**Figure 1:**
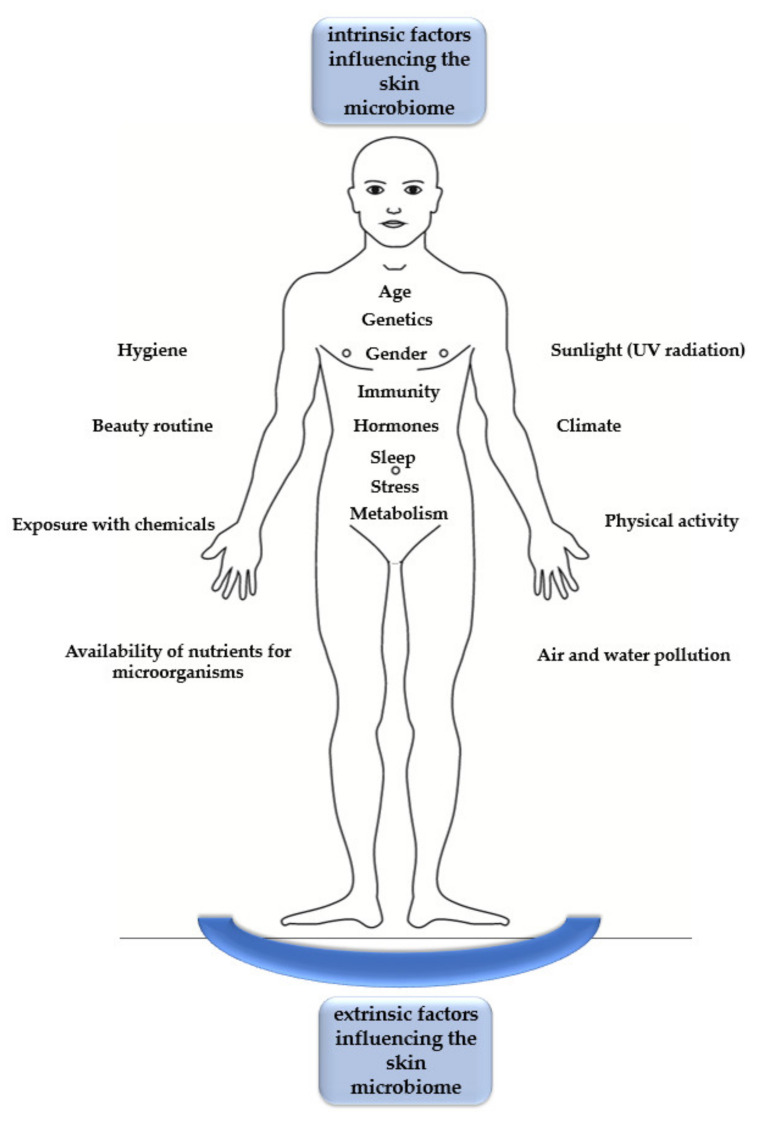
The intrinsic and extrinsic factors that influence the skin microbiome.

One factor that determines good skin functioning, is a properly working skin microbiome. This complex set of microorganisms consists of bacteria, fungi, viruses, micro-eukaryotes (mites), archaea, and phages. Probst et al. [7] observed that approximately 4% of all microbial genes are archaeal 16s rRNA genes. They showed that approximately 88% of all observed operational taxonomic units (OTUs) are composed of Thaumarchaeota species, the rest being Euryarchaeota. Dust mites are also present on human skin, being predominantly found on the face skin, around the sebaceous glands, and on hair follicles. Dust mites are found in 23–100% of healthy people and are, therefore, considered to be commensals [8]. Additionally, Demodex mites (*Demodex folliculorum* and *Demodex brevis*), associated with rosacea and chronic blepharitis, are present on the skin [8]. Phages represent an important component of the skin microbiome. They can modulate the composition and physiology of the microbial community in the microbiome [9,10]. However, there are limited data on the effect of phages on the skin microbiome due to the low phage biomass in skin samples and insufficient sequencing material [10]. The species composition, abundance, and distribution on the skin surface may vary depending on internal and external factors (Figure 1). An optimally shaped skin microbiome is the foundation of the skin’s immune system. In turn, disturbances of species composition, internal interactions, and the relationships between the microbiome and other areas of the body result in pathological changes that are not exclusively limited to the skin [6].

This review aims to discuss the latest research on the effects of various internal and external factors on the skin microbiome. The potential therapeutic applications of the latest achievements are also presented, which can prevent dysbiosis and alleviate the symptoms of the most common skin diseases.

## 2. Microorganisms Inhabiting the Skin and Methods of Their Identification

The average number of microorganisms isolated using traditional culture methods from the skin surface ranges from 10^3^ to 10^4^ CFU/cm^2^. However, in the most humid places, such as the groin, armpits, and nostrils, this value exceeds 10^6^ CFU/cm^2^. The number of microorganisms inhabiting the scalp, especially from the forehead and around the ears and head, is about 10^6^ CFU/cm^2^. On the other hand, on the upper back, chest and arms their number ranges from 10^4^ to 10^5^ CFU/cm^2^. The high number of microorganisms in hair and beards is not insignificant for the skin [3,11]. The hair shaft is constantly exposed to the environment and can be a potential site for bacteria to accumulate due to its grooved cuticle surface and long, thin structure. The bacterial community of the hair consists of Gram-positive and Gram-negative bacteria. Additionally, *Staphylococcus aureus* and *Staphylococcus epidermidis* may inhabit the human skin and scalp [2]. However, there are no data on the colonization and adherence of bacteria to hair. Studying the interaction of bacteria with human hair could be of importance in skin infections, wounds, and scalp diseases [12].

Most (>90%) of bacteria of the human skin microbiome are classified into four types: Actinobacteria (52%), Firmicutes (24%), Proteobacteria (16%), and Bacteroidetes (6%) [13]. Among them, coagulase-negative staphylococci, especially *Staphylococcus epidermidis* (10^3^–10^4^ CFU/cm^2^), anaerobic *Cutibacterium acnes* (formerly: *Propionibacterium acnes*), *Corynebacterium*, *Micrococcus*, *Streptococcus*, and *Acinetobacter* are the dominant species [3,11,14,15]. According to estimates, representatives of *Cutibacterium*, *Staphylococcus*, and *Corynebacterium* genera, isolated from almost all skin areas, may constitute 45 to 80% of the entire skin microbiome [16]. The presence of microorganisms belonging to the Archaea domain, including Thaumarchaeota, on the human skin has also been confirmed [3,15]. Research based on 16S rRNA sequencing indicates that about 4% of the microbiome genes belong to this taxon [8].

Additionally, fungi—mainly belonging to the Ascomycota and Basidiomycota types—also form part of the skin microbiome. The dominating genus is *Malassezia*. The highest level of fungi diversity has been observed on the feet, colonized by *Aspergillus*, *Cryptococcus*, *Rhodotoula*, and *Epicoccum*. However, bacteria are still the most dominant group of the skin microbiome [15,17].

Data on the presence of viruses on the human skin are scarce. This, in turn, is a natural consequence of the abundance of bacteria in the skin microbiome. Viruses belonging to the Papillomaviridae (α-HPV, β-HPV, γ-HPV), Polyomaviridae, and Circoviridae families are also commonly identified in many representatives of the studied populations, with these being characterized by high levels of variability [4,5,8].

Until recently, the identification and enumeration of microorganisms inhabiting the human skin were determined by mainly using traditional microbiological techniques. Novel approaches include molecular methods (e.g., 16S rRNA sequencing, DNA barcoding), which allow for the detection of bacteria found on the skin in low quantities, or those that are unable to grow on culture media (VBNC, viable but not culturable). Hence, these methods improve the reliability of the obtained results [2,18]. Approximately 80% of skin microbiome species are culturable [19]. Nevertheless, modern research methods allow for the identification of many new types of microorganisms that inhabit the surface and deeper layers of human skin [3,20]. One of the leading research trends in skin microbiome analysis, is the holistic treatment of this specific ecosystem without single species identification [16].

## 3. Interactions between Skin Microorganisms

The dominant resident species of skin bacteria are commensals. Together with immune cells and keratinized skin cells (replaced every four weeks), these are responsible for the appropriate skin immune barrier functioning [3,21]. There are diverse mechanisms of skin immune system support that are associated with the activity of the microorganisms. One of these is the colonization of the skin surface, preventing expansion by pathogens. Moreover, some strains of *S. epidermidis* synthesize phenol-soluble modulins (PSM), which, due to their alpha helical structure, destroy the c l membrane of pathogens. Other strains of this species can produce a lipopeptide that supports antimicrobial host defense. *S. epidermidis* can also produce lipoteichoic acid, a TLR2 ligand that plays an important role in reducing skin inflammation. The influence of these bacteria on the inhibition of the biofilm formation process by *S. aureus* has also been proven [6,22]. *C. acnes* produce lipases that hydrolyze the lipids present in sebum, releasing free fatty acids, which acidify the skin surface and create conditions that are unfavorable for colonization by pathogens from the external environment [2,23].

Additionally, AMPs (antimicrobial peptides) play a fundamental role in the skin immune defense [24]. Commensal bacteria are capable of enhancing the innate immune response of keratinocytes by stimulating AMP expression. Several types of AMPs, including human β-defensins (hBD) 1-3, cathelicidin LL-37, ribonuclease RNase-7, and psoriasin (S100A7), as well as anionic AMP dermcidin, have been recognized in the human skin [25,26].

While a properly functioning microbiome of healthy skin supports the body’s immune barrier, its transition into a dysbiosis state may lead to numerous systemic disorders. Dysbiosis, which is a disturbance of the structural and functional balance of the normal microbiome, is caused by internal and external stressors. Factors used in the fight against dysbiosis and helping to restore the balance of the skin microbiota include the use of probiotics and prebiotics. Dysbiosis alters the proportions of organisms in the healthy skin microbiome and may trigger the pathogenic potential of the commensals. Examples of diseases associated with the skin microbiome composition disturbance include acne, atopic dermatitis (AD), and dandruff [27]. *C. acnes* is, directly and indirectly, involved in the pathogenesis of the common inflammatory skin disease—acne vulgaris. The free fatty acids and the pro-inflammatory virulence factors—porphyrins, produced by these bacteria, can cause skin inflammation [23,28,29].

*S. epidermidis*, which usually colonizes human skin without any negative health effects, can cause serious illness in some people. The pathogenic nature of *S. epidermidis* depends on the skin condition and the individual properties of the bacterial strain. In some AD (atopic dermatitis) patients, escalation of symptoms may be due to increased *S. epidermidis* colonization. A similar correlation applies to *S. aureus* [5,30]. However, it is necessary to clarify whether the overrepresentation of *S. aureus* in AD is a cause or a consequence of inflammation [5,31]. Moreover, *S. aureus*—considered a pathogenic species—may reside on the skin of approximately 10–20% of healthy people as a harmless commensal [32,33].

Dandruff and seborrheic dermatitis are most commonly caused by *Malassezia* yeast, which are resident microbiota of healthy skin [34]. Stehlikova et al. [35] observed that the microbiome of patients with psoriasis contained a higher number of *Streptococcus* spp., *Malassezia* spp., and a relatively low number of *Cutibacterium*, compared to healthy individuals.

## 4. Skin as a Living Environment for Microorganisms

The skin structure, which determines the composition of the skin microbiome, is an individual trait that depends, among others, on the age, sex, and health of the host. Hygiene habits have a big influence on the skin microbiome. Additionally, the host lifestyle and environment affect the number and composition of microorganisms that inhabit the skin. The microbiome composition may alter together with a deterioration in the health condition of the host, progressive aging, or even a change of residence or profession. The physical and chemical properties of the skin influence the dominance of specific microbiota, their proportions, and their mutual relationships.

The skin surface is slightly acidic (pH around 5.6) and dry, while its temperature is lower than the inside of the body. The outer layer of the epidermis continuously releases keratinized skin cells, leading to self-renewal of the skin every four weeks. Every hour between 500 to 3000 cells exfoliate from 1 cm^2^ of skin, which means that one adult releases between 600,000 and a million or more cells per hour [11]. Since about 10% of exfoliated cells contain bacteria, this mechanism may significantly affect the microbiome composition [2,3].

The thickness of the skin, the depth and location of the folds located on its surface, and the density of hair follicles and glands are all key factors that impact the host microbiome. Smaller or larger folds and natural depressions of the skin surface, such as the navel, offer a moist niche, separated from adverse environmental conditions, resulting in such areas being readily colonized by microorganisms [23]. Additionally, the activity of sweat glands (eccrine and apocrine) and sebaceous glands can affect the appearance, or domination of particular microorganisms. The secretions released by glands affect microorganisms in different ways, creating conditions that stimulate or inhibit their development. Eccrine glands are responsible for the excretion of water and electrolytes and skin acidification, which prevents the colonization and development of microorganisms. In turn, the secretions of the apocrine glands, located, for example, in the axillary vault, are converted by microorganisms to various substances that are responsible for the specific smell of sweat [36].

The sebaceous glands ensure an optimal environment for obligatory and facultative anaerobes. The sebum secreted by such glands creates a moisturizing, hydrophobic protective layer on the skin and is the source of lipids used by microorganisms. The free fatty acids produced by hydrolysis of these lipids facilitate the adhesion of bacteria to the surface of the glands and lower the skin pH, inhibiting the growth of pathogens such as *S. aureus* and *Streptococcus pyogenes.* Some of these compounds, e.g., lauric acid and sapienic acid, have inhibitory activity against G+ pathogenic bacteria, e.g., *S. aureus*, *S. pyogenes*, or *Corynebacterium bovis* [33]. Areas of the skin with a significant number of sebaceous glands, i.e., the head, neck, and upper torso, are the optimal environment for the growth of lipophilic microorganisms. They mainly include species of the genus *Cutibacterium* (46%), staphylococci (16%), and fungi of the genus *Malassezia* [36]. These sites characterize the lowest microbiome diversity [2,3,4,6,17].

**Figure 2:**
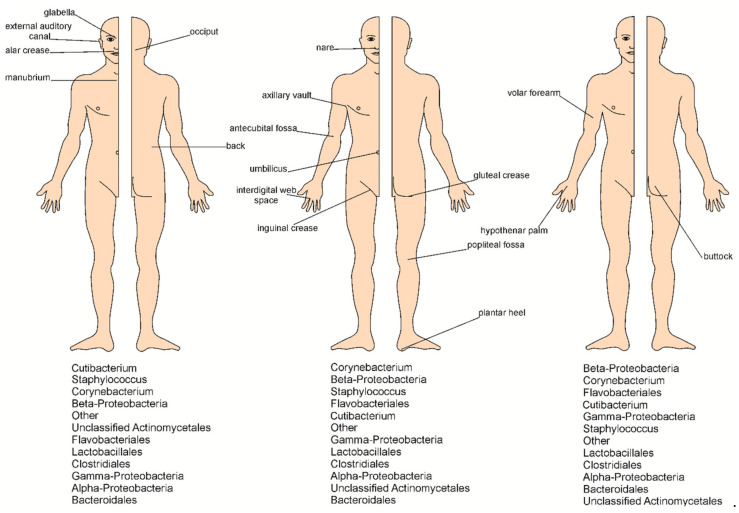
Distribution of bacteria on skin sites.

Water content is a relevant factor that influences the microbiome composition [15,22]. The human skin microbiota is diverse in different body sites (moist, sebaceous, and dry). The humid regions, such as the interdigital spaces, armpits, navel, and groin, create favorable conditions for many microorganisms and are colonized by a wide variety of species [3]. Species of the genus *Corynebacterium* (28%) and *Staphylococcus* dominate in these areas of the skin (28 and 21%, respectively) [37]. On the other hand, parts of the skin that are relatively dry and exposed to large fluctuations in temperature, e.g., the forearms and legs, are not so microbiologically diverse and contain mainly Proteobacteria (41%), Bacteroidetes (14%), and Actinobacteria (28%) [36]. The microbial and fungal diversity specific for different human body sites is presented in Figure 2 and Table 1.

The least stable, is the foot microbiome. In turn, the hand microbiota shows high stability over time, despite exposure to unfavorable environmental factors [6]. The average number of bacteria colonizing the skin of the feet ranges from 10^3^ CFU/cm^2^ on the dorsal surface of the feet to 10^7^ CFU/cm^2^ in the fourth toe cleft. In turn, the number of fungi reaches up to 80 on the sole of the heel. The most frequently isolated genera are *Malassezzia*, *Cryptococcus*, *Aspergillus*, *Rhodotorula*, *Epicoccum*, *Candida*, *Saccharomyces*, *Epidermophyton*, *Trichophyton*, and *Microsporum* [38,39]. An analysis by McCall et al. [40] showed that the fungal skin profile of human feet is correlated with urbanization. The relative abundance of *Trichosporon*, *Debaryomyces*, and *Saccharomyces* on feet decreased together with urbanization. However, the authors found the reverse correlation for the contribution of *Candida* and *Aspergillus*.

Studies on the hand microbiota have reported bacteria from four phyla: Firmicutes, Actinobacteria, Proteobacteria, and Bacteroidetes with *Staphylococcaceae*, *Corynebacteriaceae*, *Propionibacteriaceae*, and *Streptococcaceae* being the most prevalent families. *Malassezia* spp. and *Aspergillus* spp. were the most common fungi found on the palms [41].

The diversity and variability of the skin microbiome apply mainly to species inhabiting the epidermis. Meanwhile, the species composition of microorganisms of the deeper skin layers of healthy people is more universal. The number of microorganisms increases together with the content of nutrients and water in the deeper skin layers. Presumably, microorganisms that live in the dermis may determine the host immune mechanisms. However, traditional sampling methods may give the impression of an excessive dominance in the skin microbiome of migratory microorganisms and not reflect the real importance of microbiota from the deeper skin layers [41]. A better understanding of these microorganisms may consolidate and broaden the knowledge of the relationship between the skin microbiome and skin diseases [18].

## 5. The Skin Microbiome in Different Stages of Human Development

### 5.1. Prenatal Stage and Childhood

Until recently, it was believed that the interior of the uterus is sterile, and that microorganisms colonize the human skin during delivery. Meanwhile, more and more studies suggest that the uterine cavity, the placenta, and the amniotic fluid are not sterile, and the colonization occurs in the mother’s body [22,42]. The cervical mucus plug, which should guarantee sterility, allows the penetration of bacteria that inhabit the vagina. Moreover, uterine colonization may occur accidentally, e.g., during the procedures involving insertion of intrauterine devices. The bacteria isolated from the placenta include G+ and G− bacteria, including *E. coli*, *Prevotella tannerae*, and Bacteroidetes [43]. Presumably, the placental microbiome may influence the metabolism and immune response of the fetus. [22,44]. Some studies indicate that the placenta harbors a low in abundance but metabolically rich microbiome. The placental microbiome consists mainly of nonpathogenic commensal microbiota from the Firmicutes, Tenericutes, Proteobacteria, Bacteroidetes, and Fusobacteria phyla. The placental microbiome profiles were most akin to the nonpregnant human oral microbiome [45,46]. Depending on whether the delivery is via the vaginal canal or by a caesarean section, the baby comes into contact with microorganisms either in the genital tract or from the mother’s skin. Microorganisms isolated from various parts of the body of newborns—including the skin—who were born through the genital tract, belong to the maternal genus of *Lactobacillus*, *Prevotella*, and *Sneathia*. In turn, for children born via caesarean section, the skin microbiota, i.e., *Staphylococcus*, *Corynebacterium*, and *Cutibacterium* are characteristic [3,47]. Zhu et al. [48] found that the way of childbirth influences the composition of the facial skin microbiome in 10-year-olds. Some researchers suggested that the treatment of children born by C-section with vaginal secretions collected from a healthy mother before delivery, supplement their microbiome composition. The skin microbiota of children treated in this way were similar to that of newborns born through the birth canal, especially in the first week of life [49]. However, studies by Stinson et al. [50] questioned the effectiveness and safety of this procedure.

**Figure 3:**
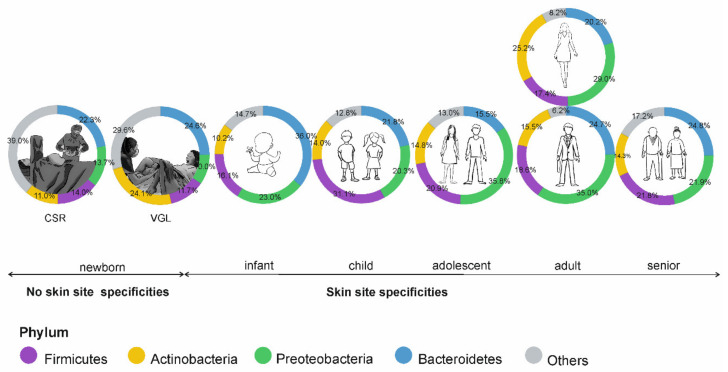
Age-dependent specificity of the skin microbiome; CSR—cesarean section, VGL—vaginal birth.

Intensive mother–child relationships in the first few months after birth lead to the transmission of microorganisms between them. Additionally, a child’s contact with environmental factors increases the species diversity of the skin microbiome. A study of the microorganisms inhabiting the body surface of 1-year-old infants, showed a dominance of bacteria belonging to the Firmicutes type (about 50%), followed by Actinobacteria and Bacteroidetes (about 20% each). In adults, the latter type is most common on the skin. Despite the specificity of the skin microbiome in infants, no typical differences in the species composition of microorganisms inhabiting the face and arms of adults were observed (Figure 3) [5,47].

### 5.2. Puberty and Adulthood

The species diversity of the skin microbiome progresses at least until the age of eight. The number of *Staphylococcus* or *Streptococcus* species decreases, and the amount of Actinobacteria and Proteobacteria species increases [51,52]. In pre-pubertal children, a greater diversity of fungal species than in adolescents and adults is observed. However, the greatest variety of fungi characterizes the skin microbiome of middle-aged people [15,52,53].

The colonization by new species is often a direct result of the appearance of specific nutrients that are metabolized by these microorganisms. In adolescents, who secret more sebum, increases in the number of *C. acnes* hydrolyzing triglycerides are observed [3]. Presumably, the stable composition of the skin microbiome is achieved together with the normalization of internal factors related to, for example, puberty [29].

Microorganisms inhabiting the human skin may impact the processes that are essential for skin aging, including the regulation of immune functions, resistance to ultraviolet radiation, and the biosynthesis and metabolism of substances related to progressive aging [52]. The effect of microorganisms depends on the age of the individual and the colonization area (Figure 3). While the presence of *Streptococcus* in the children’s skin microbiome is a positive phenomenon, the same bacteria negatively affect the skin condition of the elderly [52]. Additionally, natural changes in the structure and topography of the skin, that accompany the aging process, for example, wrinkle formation, influence the skin microbiome composition. Increased susceptibility of the elderly to infections, resulting from, for example, reduced activity of AMP, may facilitate the colonization and growth of various microorganisms, including pathogens. Together with the aging process, the number of Firmicutes, including *S. aureus* and *Cutibacterium* species, in the microbiome decreases [29,54]. Juge et al. [55] observed that the number of Proteobacteria and Acinetobacteria on the skin of women aged 54–69 was higher and lower, respectively, compared to the skin of younger women (21–31 years). On the other hand, more *Corynebacterium* bacteria and fewer *Cutibacterium* were isolated from a group of elderly women. The reduction in the occurrence of *Cutibacterium* spp. on the cheeks, forearms, and forehead of the elderly is associated with a lower level of sebum secretion [56].

## 6. Gender and the Skin Microbiome

The differences in species composition between the male and female microbiome result from sex-specific properties of the skin, i.e., the skin thickness, the number of hairs, sweat, and sebaceous glands [3,57]. The female skin microbiome is characterized by a higher species diversity that of males [58]. Presumably, a greater variety results from the thinner skin, lower pH, and less intense sweat production [59]. Sex hormones, which affect immune cell functioning, are also crucial [42]. The interactions between the microbiome, the hormonal balance, and the body’s immune mechanisms are defined as the “microgenderome” [60]. Another factor that modifies the skin microbiome is daily personal hygiene. A study of microorganisms inhabiting the surface of hands showed a greater species diversity in women than in men. On female hands, the number of vaginal microbiota, i.e., Enterobacterales and Lactobacillaceae was significantly higher (300–400%), whereas in men, higher concentrations of *Cutibacterium* and *Corynebacterium* were observed [3,41]. An important aspect is proper hand hygiene, as hands can be a reservoir of pathogens [41]. Zapka et al. [61] showed that washing hands with soap from open refillable dispensers increases the level of hand contamination with opportunistic pathogens among primary school children. However, most studies show beneficial effects of handwashing and the use of disinfectants in reducing pathogens on the hand surface [41]. The use of cosmetics can adversely affect the skin microbiome. Excessive use of cosmetics may reduce the number and variety of microbes that colonize the skin [62]. Bouslimani et al. [63] found that modification of hygiene routines can alter the skin microbiome, depending on the product used and the location on the body. Certain cosmetic ingredients may promote or inhibit the growth of certain bacteria, e.g., the lipid components of moisturizers can provide nutrients and promote the growth of lipophilic bacteria such as *Staphylococcus* and *Cutibacterium*. Disrupting the microbiome can cause inflammation, irritation, dryness, itchy skin, dermatitis and worsen the condition of the skin. Hygiene practices should reduce pathogenic microorganisms without disturbing the skin microbiome [64].

## 7. Ethnicity and the Skin Microbiome

Among the genetic factors shaping the skin microbiome, ethnicity appears to be secondary, but not insignificant. Of great importance, is the broadly understood lifestyle, including, above all, hygiene habits [3]. The study by Li et al. [57] revealed that the skin microbiome composition of East Asian people is specific. Another research study showed that the number of *Cutibacterium* on the scalp and armpits of males in Africa and Latin America is lower than in other ethnicities (Caucasian, African–American, East Asian, and South Asian). The microbiomes of the arms of African–American men and the armpits of East Asians were significantly different compared to other groups [65]. Despite some similarities, the Chinese skin microbiome differed from other populations [66]. The study of the skin of the hands of women from Tanzania and America confirmed the possible influence of ethnic factors on the microbiota inhabiting these parts of the body’s surface [67].

## 8. Living and Working Environment

Age, gender, and ethnicity are the most important factors that are specific to the individual host and affect the skin microbiome. The environment of a given individual is also extremely relevant, as well as the type of daily activity or profession. The results of a study carried out in Finland showed a significant influence of urban and rural environments on the skin microbiome of children aged 1–4 years. This effect disappeared in teenagers (14 years old), which resulted directly from the limited time this age group spent outdoors. However, the results of studies obtained in other countries did not confirm this tendency, suggesting that other factors (cultural differences) also impact the skin microbiome [51]. Different animal species contain unique microflora, often of the same or greater complexity than the human microbiome [68]. Constant contact with animals influences the composition and diversity of the skin bacterial community in healthy people [69,70]. Kraemer et al. [71] found that the microbiomes of the nose and skin of people living in a household with animals are more similar compared to those who did not live with animals, suggesting the effect of pets in promoting microbial exchange. Mosites et al. [72] showed that pig farming has a significant impact on the nasal microbiome of pig farmers. In turn, Torres et al. [73] observed that domestic dogs and their household owners share bacterial populations. The structure of the bacterial community was affected by the season, but not dog sex, age, breed, or coat type.

Differences in the skin microbiome of rural and urban residents may be associated with a different degree of exposure to microorganisms from the soil, water, and biomass used in agriculture or livestock [22]. Even short-term skin contact with soil and plant materials leads to changes in the hand microbiome and an increase in the abundance of Acidobacteria and Bacteroidetes [74]. On the other hand, city dwellers work mostly indoors and spend their time inside buildings [75]. The study by Hospodsky et al. [67] found that the hand microbiome of Tanzanian women, working outdoors and having constant contact with soil and water, was dominated by the environmental bacteria of Rhodobacteraceae and Nocardioidaceae families. In turn, the hands of female students from the United States were mainly colonized by *Staphylococcaceae*, *Propionibacteriaceae*, *Streptococcaceae* and *Xanthomonadaceae*.

Microorganisms that occur in the environment of enclosed spaces, characteristic of urban and industrial areas, come mainly from the microbiome of people living in them [40]. They include microorganisms that inhabit the skin, gastrointestinal tract, and the genitourinary system [76]. The relative abundance of fungi and bacteria associated with human skin increases along with the indoor urbanization progress. Additionally, the number of potentially pathogenic fungi, including *Aspergillus*, *Malassezia*, *Candida*, and *Eurotiales* also increases. Together with urbanization, the bacterial diversity of the skin decreases due to hygiene habits and Western lifestyles. Many skin commensals (e.g., *S. epidermidis*, *Lactobacillus* spp., *Burkholderis* spp., *C. acnes*) are lost and replaced by *Staphylococcus*, *Corynebacterium*, *Cutibacterium* and *Micrococcus* [75].

The rural environment is characterized by a high level of microbiological diversity. Research by Ying et al. [58] showed that the microbiome of people living in rural areas is more variable than urban residents, and the occurrence frequency of some species is different. Bacteria of the genus *Cutibacterium* were found more frequently on the back skin of adults living in the countryside, whereas the genus *Trabulsiella* was more abundant on the hands and forearms of urban residents. One important source of microorganisms in rural environments, is domestic and farm animals. In such an environment, antibiotic-resistant strains pose a particular threat. Human–animal interactions can change the skin microbiome composition, including declines in *Staphylococcus* and *Streptococcus* numbers.

The reductions in *Corynebacterium* and *Cutibacterium* numbers and increases in *Pseudomonas* and *Acinetobacter* amounts were observed primarily in farmworkers who were in contact with various species of farm animals [77].

The skin microbiome is affected by external environmental conditions, including temperature, humidity, and sunlight. Solar radiation, and above all UV radiation, is of special interest due to its antibacterial and destructive effect on skin cells. Intense exposure of the skin to UV radiation may increase its susceptibility to infections and exacerbate the associated symptoms, such as in the case of herpes simplex virus (HSV) [8]. On the other hand, some bacteria can protect the skin from the destructive effect of UV radiation. Li et al. [52] found that Cyanobacteria and *Lactobacillus* on the skin surface decreased the intensity of pigmentation and the occurrence of damage related to so-called photoaging.

The resistance of the skin microbiome to solar and UV radiation is varied. The commensal *Malassezia furfur* showed high sensitivity to UV radiation, despite their ability to synthesize a UV filter-like substance—pityriacitrin. Exposure of the skin to UV rays resulted in an overall increase in Cyanobacteria numbers and reductions in Lactobacillaceae and Pseudomonadaceae amounts [78]. Sunlight and UV light also effectively inhibited the growth of *S. aureus* and *C. acnes* [8]. The reduction in *C. acnes* number is associated with the decreased production of porphyrins [79].

Skin treatment with UV radiation may also affect the genetic variability of the skin microbiota and disturb the healthy microbiome structure [8].

## 9. Antibiotics

The use of antibiotics in the treatment of skin diseases is a standard procedure. Such an approach stabilizes the skin microbiome composition and reduces pathogens. The correct selection of an antibiotic determines the successful treatment, while limiting its impact on other microorganisms inhabiting the skin surface. Orally administered minocycline (used in the treatment of acne) decreased the abundance of *Cutibacterium*, *Corynebacterium*, *Prevotella*, *Lactobacillus*, and *Porphyromonas* [80]. In turn, doxycycline significantly reduced the number of *C. acnes* (1.96 times after 6 weeks of treatment). The number of *Snodgrassella alvi* also decreased (3.85-fold). On the other hand, a statistically significant increase in the number of *Cutibacterium granulosum* (4.46 times) was observed [81].

Macrolides, tetracyclines, and clindamycin are used to treat acne. Cheek skin therapy with lymecycline reduced the presence of *Cutibacterium* and increased the number of *Streptococcus*, *Staphylococcus*, *Micrococcus*, and *Corynebacterium* [82]. In turn, minocycline contributed to microbiome disorders [3]. While fluoroquinolones (perifloxacin) and macrolides (erythromycin) significantly decreased the number of *C. acnes*, inhibitory activity against coagulase-negative staphylococci was exhibited only by nadifloxacin [14].

Apart from the bacterial inhibition and reduction in inflammatory lesions, the use of antibiotics may lead to the appearance of antibiotic-resistant species in the skin microbiome, e.g., *C. acnes* and *S. epidermidis* [82]. Long-term acne therapy with macrolides increased the number of *C. acnes* isolates with reduced sensitivity to the effects of macrolides. According to estimates, the percentage of erythromycin and azithromycin-resistant strains may reach 50%, and even 100%, respectively [82,83]. Among G+ bacteria isolated from infected skin, 77.5% were resistant to penicillin and 28% to methicillin. Of all the strains tested, 31.9% were insensitive to more than three antibiotics. The results of children’s skin analyses showed resistance to methicillin in 36.4% of *S. aureus* strains isolated from the surface of the skin [84]. Furthermore, methicillin-resistant staphylococci (MRSA) are one of the most common causes of nosocomial infections [85,86].

## 10. Microbiome Transplantation—An Alternative to Antibiotics in the Treatment of Skin Diseases

One of the latest trends in research on the skin microbiome, is the development of transplantation methods for therapeutic purposes. In such treatments, the skin microbiome of a healthy individual is transferred to a washed and/or disinfected area of the skin of another person in order to improve the skin condition of the latter [87]. Studies have reported attempts to transplant the microbiome within the recipient’s body [88,89]. Paetzold et al. [90] confirmed that the application of a suspension of various components of the skin microbiome of a healthy individual changed the species composition of the recipient’s microbiome. The dominance of the donor strains lasted for about one week. Moreover, final studies did not confirm any side effects of the experiment. A disadvantage of skin microbiome transplants is the limited number of bacteria collected from the human skin. Another limitation is that we do not know exactly which bacteria, fungi, or viruses are transferred to the recipient’s skin. Hence, such an approach poses a risk of pathogen transmission [87]. Alleviation of disease symptoms was also observed after transplantation of *Roseomonas mucosa* onto the skin of people with atopic inflammation (AD) [91]. This species produces substances that control the growth and pathogenicity of *S. aureus* [29]. Genetic engineering of skin commensals (*S. epidermidis*, *C. acnes*) represents a new and promising approach for the production and secretion of active biotherapeutics (Fillaggrin, LEKTI, IL-10, anti-inflammatory somatotropin and other growth factors and hormones) [87].

Skin-derived coagulase-negative *Staphylococcus* (CNS) secrete antibacterial compounds that inhibit the growth of many G+ bacteria, including *C. acnes* [92]. Williams et al. [31] observed that *S. aureus* is susceptible to lantibiotics—synthesized by CNS—that were applied onto the skin surface of individuals affected by AD. Active strains of *S. epidermidis* and *Staphylococcus hominis*, applied to the skin surface at a concentration of 1 × 10^5^ CFU/cm^2^, significantly reduced the abundance of *S. aureus* on the skin of AD patients after 24 h [93]. Strains of *S. epidermidis* that produce substances that inhibit the microbes that cause skin inflammation, have been used in a cream to alleviate the symptoms of AD. The application of such cream caused a 90% decrease in the number of *S. aureus* on the skin after just one application and almost complete elimination after a week of use [93].

Therapy based on skin microbiome transplantation is an alternative to antibiotics. Such an approach limits the risk of developing drug-resistant bacterial strains and guarantees the patient’s skin microbiome balance [38].

## 11. Cosmetics and Clothing Textiles

Cosmetics aim to improve the quality of the skin and slow down the aging process. These products may contribute to the diversification of the skin microbiome, especially, when used regularly or over a long-term [29]. The active ingredients contained in cosmetics may favor or inhibit the growth of certain microorganisms. Among the compounds that stimulate the skin microflora is N-acetylglucosamine, a precursor of hyaluronic acid, commonly found in skincare cosmetics [29]. Bouslimani et al. [63] and Callewaert et al. [94] reported that antiperspirant and foot powder increased skin microbiome diversity. The effect vanished after stopping antiperspirant application. On the contrary, hand and face lotions did not have any major impact on microbial diversity. Moisturizers reduce the intensity of water loss from the skin and can support the skin microbiota, while decreasing skin cell exfoliation [3]. Their lipid compounds promote the growth of lipophilic bacteria, such as *Staphylococcus* and *Cutibacterium* [63]. On the other hand, the increased level of skin hydration decreases sebum content and may reduce *Cutibacterium* number [95]. Lee et al. [96] found that the application of a set of moisturizing products increased the bacterial diversity of the skin microbiome but reduced the number of *Cutibacterium*.

Additionally, bacteria can be active ingredients in cosmetics, for example, probiotic bacteria, mainly of the *Lactobacillus* genus. Their antagonistic activity against pathogens may result from the competition, synthesis, and secretion of various antimicrobial substances or blocking their adhesion to skin cells [97]. One study showed that probiotic bacteria *Lactobacillus rhamnosus*, *L. reuteri*, *L. acidophilus*, *L. delbrueckii* and *Bifidobacterium bifidum* reduced the risk of developing AD and acne and were effective in wound healing when antibiotics failed [95]. Ointment containing *L. reuteri* DSM (German Collection of Microorganisms and Cell Cultures GmbH) 17938, a strain with proven antagonistic properties against skin pathogens, positively affected AD skin [97]. However, daily application of this probiotic strain had a transient effect on the skin microbiome, and the relatively high number of the microbiome decreased after two weeks [98].

Cosmetics ingredients act for several weeks, and the reactions of individuals can vary widely [63]. Inappropriate cosmetics, or their unsuitable application, negatively influence the skin microbiome by reducing its diversity, leading to dysbiosis. Cosmetics, such as shampoos or creams, can also cause infections, sometimes leading to serious health consequences, especially when used in children or people with reduced immunity [11].

Skin-to-clothing contact is also of importance, which leads to the transmission of microorganisms and the formation of the so-called textile and volatile microbiome. In turn, the composition of the fabric microbiome is affected by washing and drying [99]. Microorganisms that adhere to fibers can use dirt or sebum compounds as a substrate and produce volatile substances as by-products, contributing to unpleasant odors [100,101].

## 12. Skin Disinfection and Its Influence on the Microbiome Condition

**Figure 4:**
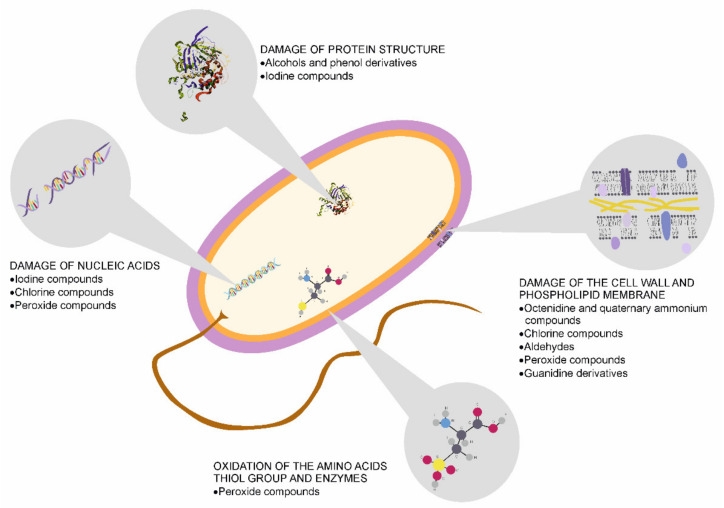
Effect of disinfectants on microorganism cells—modes of action.

In the COVID-19 pandemic (winter 2020–2021), skin disinfection, especially hand sanitation, is of great importance. Its effect depends on many environmental factors, e.g., temperature, humidity, as well as the type, concentration, and exposure time of the selected aseptic agent. The specificity and the number of microorganisms that inhabit the disinfected region, skin pH, humidity, and structure, the thickness of glands on the skin, and their secretions are also essential for successful disinfection processes [102]. Mechanisms of action of disinfectants on microorganism cell structure has been shown on Figure 4. In the hospital environment, the method of disinfectant application (peeling, swab, applicator, or ampoule) and the complexity of the disinfection procedure (single or multi-stage) play relevant roles [103].

Soaps, combined with mechanical removal, effectively reduce the number of microorganisms on the hands [14]. Kundrapu et al. [104] showed that washing the hands with soap and water significantly decreased the number of *Clostridioides difficile* spores on the hands. It also seems that water and soap are more effective than non-aqueous products in removing dirt and microorganisms from the surface of the hands [105]. Enriching traditional soaps with antibacterial substances may increase their disinfecting effectiveness. Benzalkonium chloride and triclocarban added to soap limited the growth of group A streptococci [106]. Body wash lipid formulas containing zinc pyrithione (ZPT) reduced *S. aureus* abundance, positively affecting the AD skin microbiome structure [95].

Additionally, the formulation (liquid, gel, foam) and the amount of applied disinfectant are crucial for sanitation effectiveness [107]. Too frequent use of soap or other antiseptics in hand disinfection can disrupt the microbiome and reduce its diversity due to damage to the skin surface and impairment of its protective function [14,22,86,108].

Antiseptics most commonly applied to reduce the risk of surgical site infections and bacteremia, include iodine povidone, chlorhexidine, and ethanol [109,110,111]. Chlorhexidine, present in commercial preparations in the form of chlorhexidine gluconate, shows a high affinity for the skin [103]. Its effectiveness lasts for several hours after application and is higher when combined with alcohol rather than when used in an aqueous solution. The agent was shown to be more effective than povidone-iodine alcohol solutions against short-term infections associated with catheterization [109,112]. Chlorhexidine gluconate showed comparable efficacy against *C. acnes* on the skin to isopropyl alcohol. Additional mechanical scrubbing of the skin did not change its effectiveness [113]. *C. acnes* may colonize deeper layers of the skin and become more resistant to disinfection. One active substance, which penetrates the sebaceous glands and effectively inhibits the development of *C. acnes*, is benzoyl peroxide (BPO). Its application reduced the *C. acnes* number on the shoulder skin, but not in all the tested individuals, probably due to the discontinuous treatment [114]. The combination of chlorhexidine with benzoyl peroxide, however, did not increase the inhibitory effect on *C. acnes* [115]. The study showed that some *C. acnes* isolates required a higher chlorhexidine digluconate MBCs (minimum bactericidal concentrations) than the concentration used in commercial preparations (2%). On the other hand, MBCs determined in the same study for iodine-povidone, ethanol, and benzalkonium chloride were lower than their commercial concentrations [116].

Preparations based on alcohols, primarily isopropyl or ethanol, are also frequent ingredients in skin disinfectants. Due to their low aggressiveness, even their long-term use does not affect the skin integrity [14]. Tests conducted on health care workers’ hands showed that such agents do not influence the diversity of the skin microbiome [41]. Due to the relatively low spore and virucidal effectiveness, they are often combined with other substances [102,105,107]. Despite the fact that suspension tests confirmed the efficacy of ethanol-based antiseptics against various viruses (e.g., poliovirus, adenovirus, and virus), a modified fingerpad test found better effects of a soap containing povidone-iodine [117]. Tests carried out with the same method proved that soap is better at reducing the titer of human intestinal and respiratory noroviruses than preparations based on propanol and ethanol [118]. On the other hand, ethanol (78–95%), propanol (70–100%), and iodine-povidone (0.23–7.5%) effectively eliminated coronaviruses in the suspension test. The chlorhexidine digluconate (0.02%) was inefficient. A 1 min exposure to ethanol (62–71%) reduced the coronavirus titer by 2.0–4.0 log10 [119].

## 13. Gut–Skin Axis

An important aspect is the gut–skin axis. The microbiome is the most important regulator of the immune system. Both the intestine and the skin contain various species of bacteria, fungi, and viruses that maintain a symbiosis with the human habitat. Violation of this balance can lead to impaired barrier function. The main task of the microbiome is to maintain homeostasis through two-way communication with tissues and organs. Dysbiosis of the skin or gut microbiome is closely associated with an altered immune response, accompanied by skin diseases, including atopic dermatitis, psoriasis, acne vulgaris, dandruff, and even skin cancer [120]. Diet and lifestyle primarily influence the proper composition of the intestinal microbiome. Disturbances in the intestinal microbiome lead to dysfunctions, such as rheumatoid arthritis, psoriasis, and atopic dermatitis [121]. Celiac disease and gluten sensitivity are also associated with skin lesions, including dermatitis herpetiformis, and psoriasis. These changes disappear after switching to a gluten-free diet [122]. A similarly strong link between atopic dermatitis and food allergy indicates the importance of food underlying the gut–skin axis [123]. The exposure of the skin to external factors, e.g., ultraviolet B (UVB) radiation, also influences the intestinal microbiome [124]. Food allergies can result from the impairment of the skin barrier, e.g., atopic dermatitis increases the risk of developing a peanut allergy. This allergy results from the epidermal exposure to a peanut protein contained in house dust, leading to immunoglobulin E-mediated mast cell expansion in the gut (IgE) [125,126]. In recent years a large number of articles have focusing on the gut–skin axis have been published. However, there are still knowledge gaps in this area, and this topic merits further investigation.

## 14. Summary

The skin microbiome plays a relevant role in maintaining the proper functioning of the human organism. Currently, there is growing interest in skin microbiome research. Researchers have been concentrating on the development of more accurate methods of microbial testing. Such techniques enable the identification of new microorganisms that inhabit both the surface and deeper layers of the skin. Studies of the influence of environmental factors on the skin microbiome also represent a popular research direction. Of great interest, is the role of the skin microbiome in the regulation of host immune mechanisms.

Studies on the skin microbiome allow for a better understanding of the interactions between microorganisms and the impact of the external environment on the microbiome balance. In turn, functional studies allow for the development and optimization of therapeutic methods that enable the inhibition of skin pathogen growth.

## Figures and Tables

**Table 1:** Composition of the human skin micobiota in various locations.

		Skin Sites			
		Moist	Sebaceous	Dry	Foot
**Body sites** **location**		groin, axilla, antecubital fossa, toe web	face, chest, back,	arm, leg, volar forearm	Moist—toe interdigital web space dry—plantar heel
Fungi	*Malassezia* spp.	36% CH–80%AD	65% CH–99%AD	35% CH–83%AD	53–80%
	Other fungi	Ascomycota: *Aspergillus*, *Epicoccum*, *Phoma* (levels >5%): 9.5%AD–40.2%CH *Cladosporium*, *Cryptococcus*			*Cryptococcus*, *Aspergillus*, *Rhodotorula*, *Epicoccum*, *Saccharomyces*, *Candida*, *Epidermophyton Microsporum*, *Trichophyton*

CH—children, AD—adults.
